# Preclinical Imaging Biomarkers for Postischaemic Neurovascular Remodelling

**DOI:** 10.1155/2019/3128529

**Published:** 2019-02-03

**Authors:** Richa Gandhi, Charalampos Tsoumpas

**Affiliations:** Department of Biomedical Imaging Science, Leeds Institute of Cardiovascular and Metabolic Medicine, University of Leeds, Leeds LS2 9NL, West Yorkshire, UK

## Abstract

In the pursuit of understanding the pathological alterations that underlie ischaemic injuries, such as vascular remodelling and reorganisation, there is a need for recognising the capabilities and limitations of in vivo imaging techniques. Thus, this review presents contemporary published research of imaging modalities that have been implemented to study postischaemic neurovascular changes in small animals. A comparison of the technical aspects of the various imaging tools is included to set the framework for identifying the most appropriate methods to observe postischaemic neurovascular remodelling. A systematic search of the PubMed® and Elsevier's Scopus databases identified studies that were conducted between 2008 and 2018 to explore postischaemic neurovascular remodelling in small animal models. Thirty-five relevant in vivo imaging studies are included, of which most made use of magnetic resonance imaging or positron emission tomography, whilst various optical modalities were also utilised. Notably, there is an increasing trend of using multimodal imaging to exploit the most beneficial properties of each imaging technique to elucidate different aspects of neurovascular remodelling. Nevertheless, there is still scope for further utilising noninvasive imaging tools such as contrast agents or radiotracers, which will have the ability to monitor neurovascular changes particularly during restorative therapy. This will facilitate more successful utility of the clinical imaging techniques in the interpretation of neurovascular reorganisation over time.

## 1. Introduction

**Figure 1:**
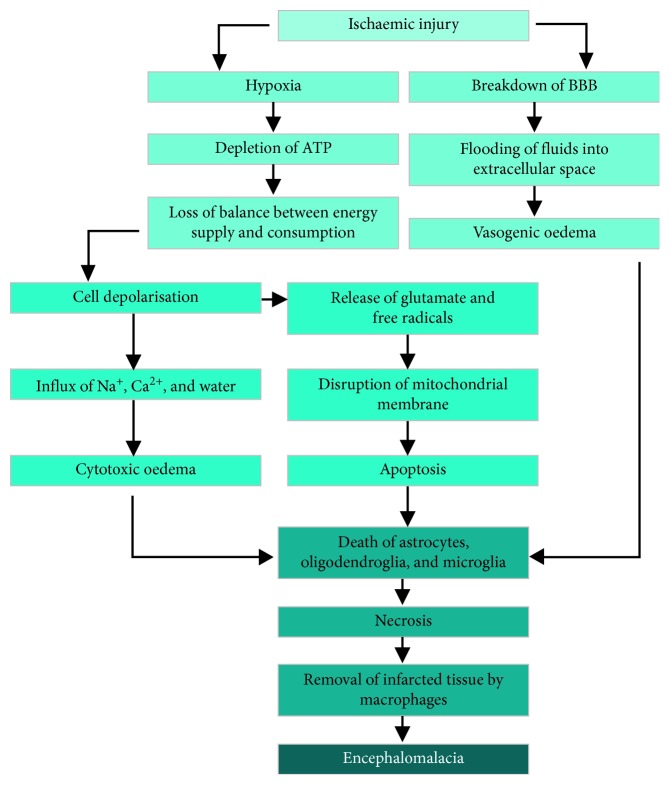
Ischaemic cascade. The cascade involves a series of events that follow ischaemic injury to the brain, such as that due to stroke. Eventually, this results in the softening or loss of brain tissue (i.e., encephalomalacia). BBB, blood-brain barrier; ATP, adenosine triphosphate.

Oxygen is essential for the survival and basic functioning of biological systems. A stable equilibrium between the supply and usage of oxygen in biological systems generates oxygen gradients, wherein areas with low concentrations of oxygen (i.e., hypoxic regions) are situated relatively distant from the central vasculature. These oxygen gradients contribute to a healthy and functioning physiology, and their disturbance—for example, due to injury or disease—can confer debilitating effects on the functioning physiology [1]. Neural ischaemia is typically a hypoxic phenomenon, usually the result of some kind of traumatic injury or vascular disruption, developing from a deficiency in blood flow to brain tissue. This interrupted circulatory flow then triggers changes in the brain's metabolic processes [2]. Immediately following ischaemic injury, a series of biochemical reactions ensue within the brain, which are collectively termed the ischaemic cascade. The ischaemic cascade involves biochemical energy depletion and the inability to maintain ionic gradients across cell membranes due to the restriction in blood supply. This eventually triggers cytotoxic and vasogenic oedema, as well as apoptotic and necrotic processes, which ultimately result in the deterioration of healthy brain tissue [3–5]. This cascade is elicited in patients who experience ischaemic stroke (Figure 1) [6].

**Figure 2:**
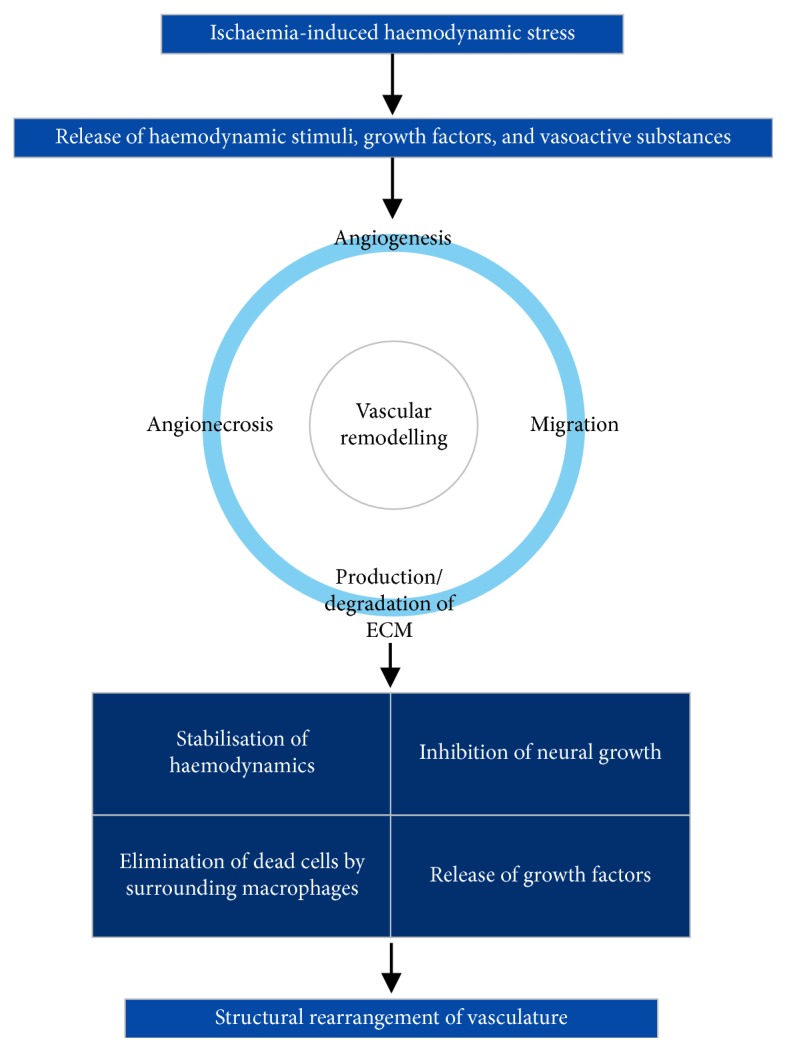
Biological process of vascular remodelling. Angiogenesis, migration of vascular cells, production and degradation of the ECM, and angionecrosis constitute the major pathological hallmarks of vascular remodelling. ECM, extracellular matrix.

One of the outcomes of an ischaemic stroke is the structural alteration of blood vessels to accommodate for the changes in haemodynamic conditions. This remodelling of the vasculature involves alterations in angiogenesis, the development of new blood vessels from preexisting ones; angionecrosis, the death of blood vessels or of the walls of blood vessels; migration of cells that compose blood vessels; and production and/or degradation of the extracellular matrix. Essentially, vascular remodelling is dependent on an interplay of haemodynamic stimuli, growth factors, and vasoactive substances [7]. As a result of the inclusion of a wide range of processes, it is essential to briefly address that the terminology of vascular “remodelling” may be used interchangeably with “alterations,” “plasticity,” “reorganisation,” “rearrangement,” or other similar keywords in the literature. The biological process involving this interplay is described in Figure 2, wherein the detection and relay of haemodynamic signals ultimately lead to vascular reorganisation.

Knowledge of the overall neurovasculature is useful for distinguishing the blood vessel or group of vessels that may be associated with an ischaemic stroke; unusual vascular patterns can then be attributed to other diseases, such as seizures, intracerebral haemorrhage, or brain tumours [8]. The functional deficits resulting from an ischaemic stroke are dependent on the cerebral arteries—and thus the brain regions—that are disrupted [6]. The cerebral hemispheres are each supplied by the posterior, middle, and anterior cerebral arteries. The posterior cerebral arteries originate from the basilar artery and supply the medial and posterior regions of the temporal and occipital lobes, as well as the thalamus and brainstem regions. The middle cerebral arteries, which originate from the supraclinoid internal carotid, supply the anterior and lateral regions of the temporal lobes and lateral regions of the parietal and frontal lobes. The anterior cerebral arteries also originate from the supraclinoid internal carotid and supply the anterior regions of the basal ganglia, as well as the medial regions of the parietal and frontal lobes [9].

Imaging techniques play a significant role in clinical research; they are crucial for enhanced visualisation to better understand biological processes. The world's first images of human brain metabolism were acquired via continuous inhalation of positron-emitting radioactive oxygen ([^15^O]) to study the neurovascular distribution and accumulation of oxygen [10, 11]. Since then, our understanding of neurovascular dynamics has significantly progressed; various neuroimaging techniques with additional advantages, such as improved spatial resolution and the capacity for longitudinal imaging, are implemented in preclinical research and clinical practice. Neuroimaging (i.e., the use of imaging for neurological structures) can serve two purposes: (1) to differentiate amongst various morphological structures and (2) to visualise functional changes in neural plasticity following injury [6]. One such example involves imaging patients immediately after the onset of a stroke and posttraumatically at regular intervals. It is well established that patients who experience ischaemic stroke subsequently undergo vascular remodelling within the brain [2, 5, 7]. Studies of stroke in humans are limited because of the difficulties in collecting postmortem tissue samples when neuronal death occurs. Thus, neural ischaemia research is largely built upon information gathered from studies of animal models. When ischaemia is induced in experimental rodent models, for example, blood flow is disrupted to restrict the delivery of oxygen and glucose to neurons, eventually causing a depletion in biochemical energy [1, 12].

The aim of this review is to provide a guide to current imaging techniques being used to explore neurovascular remodelling in a preclinical in vivo setting. This review will contribute to the following:

- Identify and distinguish preclinical imaging options used in the last 10 years
- Compare these techniques across common parameters
- Explore the potential applications of novel advances in imaging technologies
- Discuss how these imaging techniques could be translated to improve the medical management of ischaemic stroke

## 2. Materials and Methods

### 2.1. Search Strategy

The PubMed® and Elsevier's Scopus online databases were used to retrieve journal papers. Titles, keywords, and abstracts were scanned for the search terminology using the search engines. The citations and references of any retrieved papers were also scanned for any relevant literature that may have been missed in the initial search. Boolean operating search strings were employed to ensure that the most suitable papers were retrieved.  Table 1 outlines the search strategy that was implemented for literature retrieval.

### 2.2. Eligibility Criteria

Applying inclusion and exclusion parameters to the literature search process facilitated the retrieval of the most appropriate papers. Search results were restricted to papers published in English within the last 10 years (i.e., between 2008 and 2018, inclusive), as the translation of papers published in non-English languages may have distorted the understanding of the research. In vivo studies of small animals were included, and several papers discussing the same imaging technology were not disregarded, as these would only strengthen any common conclusions formed from these studies. Only primary research papers were included in the final analysis; other secondary or tertiary publications such as reviews, books, conference proceedings, presentations, and abstracts were only referred to for background or follow-up information and are cited accordingly in the discussion.

### 2.3. Quality Appraisal

The Preferred Reporting Items for Systematic Reviews and Meta-Analyses (PRISMA) guidelines were followed to ensure that the most appropriate research papers were included in this review. The PRISMA guidelines were established by David Moher and colleagues to conceptually represent the progressions within the field of systematic literature reviews. The papers that were retrieved from the search process following this model were systematically categorised according to their significance in helping to answer the overall aim of this research [13].

### 2.4. Data Collection and Analysis

Each of the retrieved papers from the literature search focussed on one or more imaging techniques. The next step involved assessing these imaging techniques based on relevant parameters to evaluate whether they could be implemented to observe neurovascular remodelling following ischaemic injury. Accordingly, the following details were extracted from each of the retrieved papers, where available: imaging technique, purpose of the study, animal model used, spatial resolution or field of view, duration of scanning, contrast agent or molecular probe utilised, safety considerations for implementation in humans, and considerations for modality-specific image interpretation.

Although an effective imaging technique may offer excellent image resolution with minimal patient invasiveness, it may not be considered optimal for the purposes of clinical translation if the acquired data are exceedingly difficult and time-consuming to acquire and even process. By analysing and discussing comparisons between different technical characteristics and across a variety of imaging techniques, appropriate strategies to employ for postischaemic imaging can then be determined.

## 3. Results

**Figure 3:**
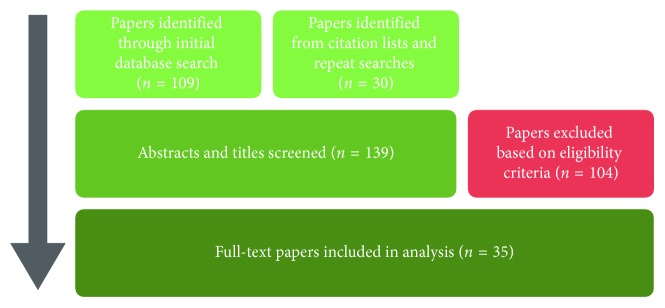
The Preferred Reporting Items for Systematic Reviews and Meta-Analyses model, as implemented for this systematic review. A final total of 35 papers were included in the literature analysis (adapted from [13]).

The PRISMA model, as outlined in the previous section, was used to select the literature for this review. The articles' filtering process is presented in Figure 3.

The results of the literature search essentially revealed a number of imaging parameters for each imaging technique that may be relevant for the imaging of neural vasculature, particularly following ischaemic trauma. These relevant parameters, along with a brief overview of the imaging techniques, are presented in Table 2.

**Figure 4:**
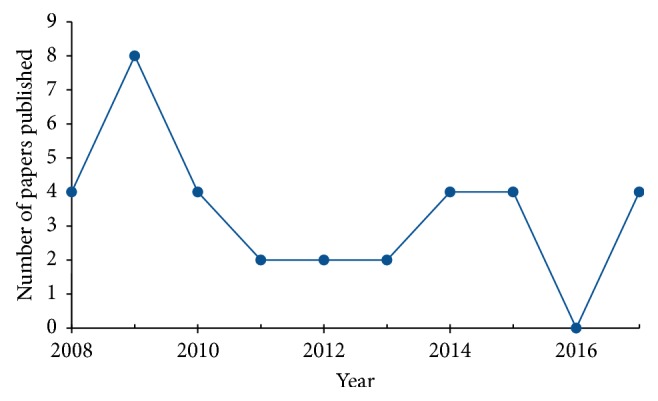
Number of publications fulfilling the search criteria per year.

The distribution of the selected publications per year is presented in Figure 4. There evidently appears to have been peaks of relevant research published in 2009, 2014–2015, and 2017 in relation to the search queries for this review, which may indicate a marginally increasing trend in studies of postischaemic neurovascular remodelling in the last 5 years.

## 4. Discussion

### 4.1. Rationale

Neurovascular remodelling is a process that establishes an ideal biological environment for neurological healing. Proceeding after an ischaemic injury, neurovascular remodelling acts to restore the haemodynamic stability of the brain, inhibit neural degeneration, eliminate any dead cells by surrounding macrophages, and facilitate the release of growth factors. Many signalling compounds and chemokines are involved in cell–cell interactions to facilitate attempts to salvage surviving neural tissue and reorganise the vasculature following ischaemic injury [17, 49]. A better understanding of these remodelling processes can enhance clinical diagnostics and restorative therapy options; thus, improved visualisation of this vascular reorganisation within the brain is beneficial. These biological pathways involve some of the same molecules that have already been used to inform the development of probes and tracers for the clinical study, such as the glucose analogue [^18^F]FDG. Nonetheless, there remains a knowledge gap about the biological processes of neurovascular remodelling that can be answered by imaging the regions in which the neurovascular changes occur. Consequently, this review purports itself to identify current imaging technologies being used in neurovascular research to observe postischaemic neurovascular remodelling in small animals. Various techniques were revealed in the published literature and evaluated against a set of properties and common measures in image analysis that were deemed relevant for vascular imaging in the brain. The imaging techniques have been categorised to help identify when certain modalities may be more appropriate for the given purpose. This review provides guidance in the identification of imaging modalities for different translational applications.

### 4.2. Animal Models of Ischaemia

**Figure 5:**
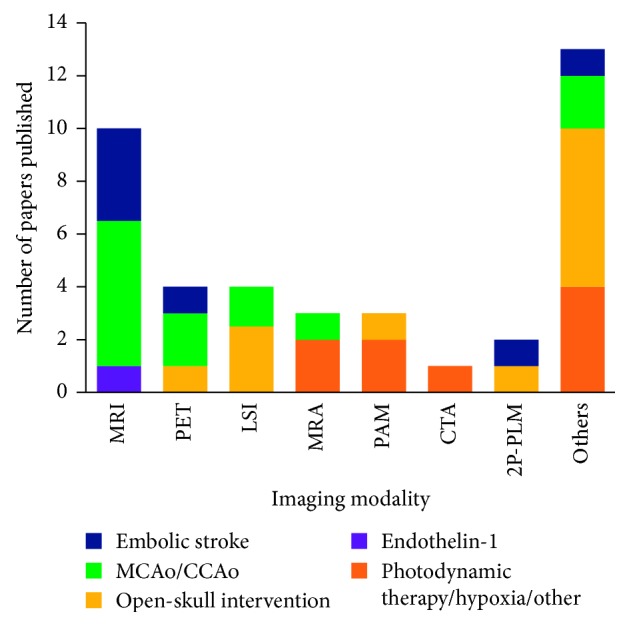
Frequency distribution of imaging modalities amongst papers retrieved through the literature search, with further distinction of the ischaemia induction models implemented. Magnetic resonance imaging and positron emission tomography were the most common techniques employed for in vivo preclinical studies of postischaemic neurovasculature. Other modalities included one instance each of multiphoton luminescence, optical coherence tomography, ultrafast ultrasound localisation microscopy, synchrotron radiation phase contrast imaging, digital subtraction angiography, microcomputed tomography, red-green-blue reflectometry, single-photon emission computed tomography, functional ultrasound, laser scanning confocal microscopy, three-photon fluorescence microscopy, and two-photon laser scanning microscopy. The MCAo/CCAo model was used most commonly across all the imaging modalities. Open-skull intervention included craniotomy, thinning of the skull, and electrode or optic fibre implantation. MRI, magnetic resonance imaging; PET, positron emission tomography; LSI, laser speckle imaging; MRA, magnetic resonance angiography; PAM, photoacoustic microscopy; CTA, computed tomography angiography; 2P-PLM, two-photon phosphorescence lifetime microscopy; MCAo, middle cerebral artery occlusion; CCAo, common carotid artery occlusion.

Small animals play a valuable role in elucidating the mechanisms underlying ischaemia. The use of small animal models allows for greater control and reproducibility to precisely evaluate ischaemic pathophysiology, compared to human cases that involve heterogeneous manifestations and causal factors. Invasive techniques can also be implemented in small animals for direct access to the brain vasculature, although they are undesirable in the context of clinical translatability. Furthermore, animal models allow for the study of immediate postischaemic changes, providing insights into early-stage mechanisms [50]. Some common rodent models of stroke include the (a) middle cerebral artery (MCA) occlusion (MCAo) model, which involves temporarily or permanently disrupting blood flow in the MCA and associated vascular branches that are predominantly affected in human stroke; (b) craniotomy model, which involves craniectomy and removal of a section of dura mater to access the MCA; (c) embolic stroke model, which involves the application of micro/macrospheres or clots to induce ischaemic lesions; (d) endothelin-1 (ET-1) model, which involves the application of ET-1 to a neural region of interest (often the MCA) to act as a vasoconstrictor and thus induce ischaemia; and (e) photothrombosis model, which involves targeted photo-oxidation to generate precise ischaemic lesions [50–54]. The most commonly reported ischaemia models in the papers identified in this review were the embolic stroke, MCAo, and craniotomy models (Figure 5). Different stroke models have different advantages and may provide information on different aspects of postischaemic changes; therefore, it is useful to consider the type of information that is desired when designing an imaging study.

### 4.3. Fundamentals

Neurovascular remodelling undergoes several phases in its process of ultimately establishing fully functional vasculature. To better understand postischaemic changes, studying neurovascular remodelling in its individual stages is ideal. The individual characteristics of these phases can be measured to provide a better understanding of the postischaemic reorganisation that occurs. Firstly, a cellular cascade instigated by endothelial cells causes heightened permeability of vessels due to the deterioration of key vascular junctions. This, in turn, enables the extravasation of plasma proteins, which helps construct a framework for the development of new vessels. Thereafter, the growth of new vasculature leads to fluctuations in haemodynamic properties, such as cerebral blood flow and volume, as well as in vessel density, whilst the vascular network continues to develop. The remapping of the vascular network in response to ischaemia is an important aspect of appreciating neurovascular outcomes [55, 56].

Therefore, it might be of considerable value to utilise small nanometre-scale intravascular contrast agents that could trace this extravasation to signal the generation of new vasculature. Agents of different sizes could help detect different levels of permeability in different areas at different intervals, whilst enhanced opacification could ideally be achieved by utilising a higher density contrast. Ideally, an imaging approach would enable the dynamic visualisation of both large and small vessels as postischaemic angiogenesis proceeds for a more complete picture of the vascular microanatomy, although studying microvascular changes might offer significant information about the earliest postischaemic processes. Hence, to distinguish individual vessels, the image resolution should complement the range of vessel diameters. An imaging resolution of at least 0.5 mm would confer the greatest benefit if the biological tracer being utilised is nonspecific to the vasculature, since cerebral arteries are larger in diameter than this, although a more precise range may be necessary to observe the remodelling of microvessels [57–59]. Nevertheless, neurovascular remodelling is not a one-off event; thus, the capacity to obtain repeated measurements over time (i.e., longitudinal imaging) is also desirable in an ideal imaging approach, thereby circumventing the need for more invasive methods. By visualising early processes, studies can infer postischaemic remodelling changes from an earlier time point. This would be valuable in a twofold manner. Firstly, experiments could retain a larger sample size, given the survival durations after ischaemia. Secondly, studying earlier fundamental processes using imaging would allow for better correlations with later outcomes in studies of neurovascular outcomes.

### 4.4. General Overview of Imaging Modalities

A wide array of imaging techniques have been implemented within the last 10 years in preclinical small animal models specifically for in vivo visualisation of vascular changes following ischaemic injury. Magnetic resonance imaging (MRI) and positron emission tomography (PET) remain the predominant modalities of choice to study postischaemic neurovascular changes, despite the advent of numerous other modalities being implemented over the last 10 years (Figure 5). According to a survey (*n* = 173) conducted amongst UK and non-UK university researchers and UK National Health Service personnel, MRI is amongst the topmost researched areas [60].

**Figure 6:**
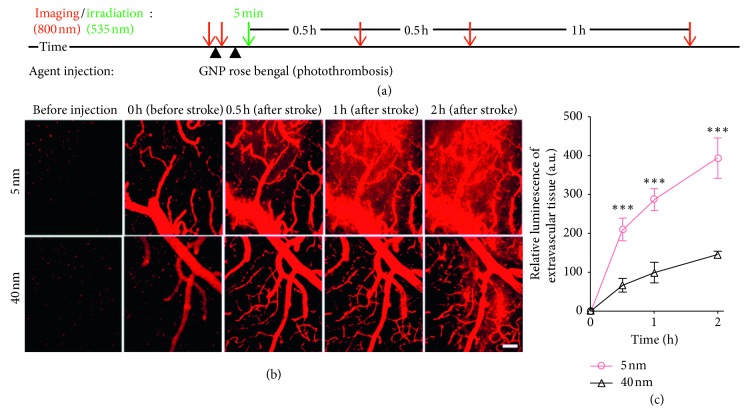
In vivo multiphoton luminescence imaging using PEG-GNPs of cerebral vasculature in murine stroke models. (a) Experimental timeline, prior to which an open-skull cranial window was generated in a live mouse. Photothrombosis was induced within the vessels to generate the mouse model. (b) Multiphoton luminescence images of neurovasculature before and after induction of photothrombotic stroke with 5 and 40 nm sized PEG-GNPs. Scale bar, 100 *µ*m. (c) Relative luminescence of extravascular tissues following photothrombosis. ^*∗∗∗*^*P* < 0.001, one-tailed *t*-test, *n* = 3; values are mean with standard deviation. PEG-GNP, polyethylene glycosylated gold nanoparticle (republished with permission from the Royal Society of Chemistry [46]; permission conveyed through Copyright Clearance Center, Inc).

Well-established modalities such as computed tomography (CT), MRI, and PET were expectedly found in the literature; in addition, several other methods have been developed and introduced relatively recently. These include ultrafast ultrasound localisation microscopy (uULM), which incorporates the use of inert gas microbubbles for precise visualisation of cortical vessel branching; synchrotron radiation phase contrast imaging (SR-PCI), which does not require the administration of a contrast agent, but it involves high doses of ionising radiation; the use of gold-coated nanoparticles in multiphoton luminescence to allow for repeated imaging over time (Figure 6); and functional ultrasound (fUS), which can be implemented in freely moving models without the use of contrast agents [16, 20, 46, 48]. Each new imaging modality improves on singular aspects of imaging vascular disease and may likely serve as an adjunct to existing clinical imaging modalities. Given the general accessibility of PET and MRI and their preexisting use in patients, these well-established techniques are likely to persist and receive the bulk of the relevant research focus. Nonetheless, much like PET/CT being a widely available hybrid system, continued study of neurovascular remodelling is likely to rely on more than one technique to develop our understanding and help fine-tune the output data from these large-scale techniques. These novel methods show promise for neurovascular imaging, and it would be of interest to follow any future work involving these methods.

### 4.5. Imaging Specifications Representing Limitations to Overcome

Multiphoton microscopy is a fluorescence technique that effectively merges laser scanning microscopy with pulsatile multiphoton excitation to image tissues that have been labelled with fluorophores [61]. This approach has been actively employed to investigate the mechanisms of neurovascular reorganisation following ischaemic injury [62]. Despite the high spatial resolution from targeted imaging, this technique suffers from slow imaging times, limited fields of view, and depth of tissue penetration. For example, live imaging in small rodents requires a cranial window—an opening in the skull for optical access to the parenchyma—to be created in the model being tested. This functionally limits the field of view available. In rats, a large cranial window (e.g., 4 × 6 mm^2^ in size) is usually created, involving removal of the overlying bone and resection of the dura mater, to enable the insertion of devices, such as cannulae and electrodes. This step is complicated by the need to reseal the window to reestablish intracranial pressure and reduce motion artefacts [63]. Mice, on the contrary, are a good alternative because cranial window generation is less invasive than that in rats; the thin skull of mice can remain intact and only requires thinning for a resulting cranial window (e.g., 2 × 2 mm^2^ in size), which produces limited disruptions in intracranial pressure and decreased inflammation [64]. Therefore, animal choice forms a key part of designing such imaging experiments.

PET and single-photon emission computed tomography (SPECT) are molecular imaging techniques that track biological target-specific changes in function with high sensitivity. Nonetheless, their relatively poor resolution (around 0.8 mm for PET and 0.3 mm for SPECT) prevents anatomically specific imaging and presents as a limiting factor, which may also augment partial volume effects and influence the measurement of vascular parameters. Partial volume effects are a direct response to multiple tissue types present within a single voxel, hence generating image blurring. Partial volume effects refer to the difference between actual and obtained image intensity values and typically occur when more than one type of tissue is contained within individual voxels, ultimately influencing the quantification of key parameters. Larger voxels have a greater probability of containing various tissue types in comparison to smaller voxels. The consequential blurring effects are essentially the result of the interplay between low spatial resolution and limited tissue sampling. Methods to correct for partial volume effects in PET imaging represent an active field of research [65–67]. The biggest advantage of using these nuclear medicine techniques despite the poor anatomical definition is that dynamic, real-time imaging becomes possible. Dynamic imaging offers considerable diagnostic information that may not be accessible from static imaging by overcoming the bias presumed by static imaging, in which a single time frame is selected to reflect the overall tracer metabolism without correcting for variabilities in distribution and activity [68]. This advantage of dynamic imaging is important for neurovascular studies when studying absolute blood flow dynamics and the remodelling process.

If anatomical detail is required, MRI provides a complement of both real-time imaging and high spatial resolution without the use of ionising radiation [3]. Access to MRI systems incorporating ultrahigh magnetic fields (i.e., greater than 9.4 T) can allow for the acquisition of images with higher spatial resolution [69]. This enhancement of resolution, however, comes at the cost of increased scan durations. With lengthier scans, the probability of involuntary movements (e.g., cyclic respiration) increases, thus resulting in lower-quality images [57]. An apparently missing imaging technique from the preclinical imaging investigations is functional MRI (fMRI). In patients, these limitations in resolution and real-time imaging have been increasingly overcome by fMRI. Even if this is perhaps one of the most successful imaging modalities in the clinic, its fundamental principle of operation and practical aspects make it largely incompatible for standard imaging of small animals [70].

**Figure 7:**
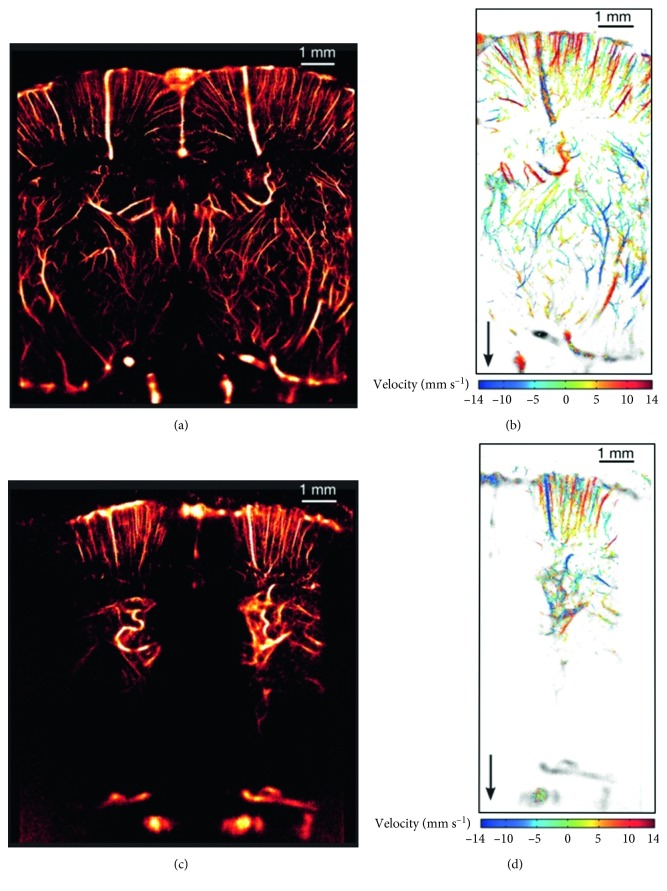
(a) Coronal-view image of uULM performed through a thinned-skull window, conferring a resolution of 10 × 8 *µ*m^2^. (b) In-plane velocity map of some vessels from (a). (c) Coronal-view image of uULM performed through an intact skull, conferring a resolution of 12.5 × 1 *µ*m^2^. (d) In-plane velocity map of some vessels from (c). uULM, ultrafast ultrasound localisation microscopy [20] (reprinted with permission from Copyright Clearance Center, Inc. [20]).

In general, particularly high-resolution parameters for vascular imaging may be achieved by the newer uULM (Figure 7) and SR-PCI techniques (<10 *µ*m), but also by SPECT (<1 mm), photon excitation microscopy (<1 *µ*m), and angiography (<20 *µ*m); however, the applications of these may come at the cost of longer scanning durations, the use of contrast agents, cranial window generation, or high ionising radiation doses [20, 21, 28, 30, 48, 62]. On the contrary, when a technique is minimally invasive to the subject, the resolution is compromised, as noted with MRI (0.1 mm, <30 mm) and functional photoacoustic microscopy (fPAM; <30 *µ*m) [14, 15, 19, 33, 37, 40, 42, 71, 72]. Researchers have many imaging modalities available to them; however, it is important to understand the limitations of each technology and design experiments based on the limitations of the modalities.

### 4.6. Contrast Agents and Molecular Probes

Imaging with molecular probes is useful for studying active biological processes at cellular and subcellular levels. Most molecular probes are radionuclide tracers and are used because of their ability to emit radiation from within the body including cells in which these tracers are taken up. As a result, radionuclide imaging offers both qualitative and quantitative data on dynamic biological processes [17, 73]. Meanwhile, contrast agents also emit signals that can enable the collection of qualitative data on the vascular architecture, although there have been discussions regarding their potential side effects [74, 75].

PET is well established as a valuable method to visualise the interplay between changes in cerebral blood flow and the metabolic needs of ischaemic tissues. The effectiveness of PET in understanding different aspects of postischaemic vascular processes depends largely on the types of tracers utilised. For example, [^15^O]-radiolabelled tracers offer the ability to quantify a number of haemodynamic characteristics, such as cerebral blood flow, cerebral blood volume, cerebral metabolic rate of oxygen, oxygen extraction, and energy metabolism [3]. The most common tracer used in PET imaging is [^18^F]FDG, although other tracers such as [^18^F]MISO, [^18^F]BCPP-EF, and [^11^C]PK11195 have been utilised as well for different applications [22, 40, 44, 76].

Different molecular factors, such as vascular endothelial growth factor (VEGF), trigger angiogenesis as a component of vascular remodelling, and these processes are regulated by the interplay between components of extracellular matrices and adhesion molecules. VEGF, associated adhesion molecules (e.g., integrins), and microvessel density can thus be useful targets to detect and visualise angiogenesis using probes that are specific to radionuclide imaging techniques [17, 73, 77]. The use of [^15^O]H_2_O as a PET tracer was also noted. Although blood oxygen level-dependent fMRI confers better spatial and temporal resolution and reduced radiation exposure, [^15^O]H_2_O PET benefits include quantitative data output and the capacity for longitudinal imaging [78]. Accordingly, few [^15^O]H_2_O PET studies have been conducted to evaluate cerebral blood flow and other haemodynamics in small animals primarily due to the short half-life of ^15^O and its potentially high-energy emitted positron, which render the PET images noisier and of lower resolution. Nevertheless, the development of [^15^O]H_2_O-based imaging in small animals is an increasing focus of recent research, and the aforementioned challenges may be technically addressed in the near future (more information is available on the website of the project Small Animal Fast MRI Insert at https://safir.ethz.ch) [43, 79–82].

Despite the high resolution of MRI, it generally does not require intravenous contrast agent injections; however, MRI may be enhanced by effective contrast agents for visualisation or quantitative analysis, and this can be facilitated by monocrystalline iron oxide nanoparticles (MIONs). MIONs are useful contrast agents because they are relatively cost-effective to produce, nontoxic, biocompatible, and both chemically and physically stable [83]. Although they are able to depict the vascular architecture clearly, it is important to note that utilising MIONs or any intravascular MRI contrast agents may cause difficulties in evaluating vessels with large diameters due to the contrast agent particles reducing the baseline intravascular signals from larger vessels. This suggests that there may exist an upper threshold in studying vascular remodelling using contrast-enhanced MRI and that only small-vessel changes may reliably be observed. This might be an important aspect to consider when assessing vascular remodelling that includes larger vessel sizes and densities [15, 34, 57, 84].

CT methods, such as micro-CT (*µ*CT) and CT angiography, offer detailed images of the neurovascular architecture. Their strengths are reflected in their ability to rapidly generate high-resolution images at relatively low costs [9, 85]. CT angiography involves the administration of an intravenous contrast agent to visualise the vasculature on thin-section images in any plane. Moreover, these detailed images can be obtained in short time frames of approximately less than a minute [21, 39]. Whilst CT does not offer the same degree of soft-tissue resolution as MRI, CT scans are not contraindicated when metal is present. This is uniquely beneficial because animals with implants or devices can still be imaged. For qualitative data on structural changes in the neurovasculature, CT may be a more practical choice of imaging modality, which may require supplementation by other imaging modalities for quantification.

This systematic literature review also revealed a number of techniques that do not require the administration of contrast agents or tracer molecules; these include fUS, photoacoustic microscopy (PAM), fPAM, laser speckle imaging (LSI), and SR-PCI [16, 32, 33, 36, 42, 48]. Evidently, evading the need for intravenous injections would render these techniques as attractive and ideal for translation to clinical practice. LSI has already been implemented to assess systemic microvascular function in patients with and without cardiovascular disease [86]. Meanwhile, PAM has been used previously to evaluate vascular profiles and oxygen saturation in patients with complex regional pain syndrome, as well as to visualise the microvasculature and changes in vascular networks in patients with and without acral melanoma [87, 88].

### 4.7. Safety Considerations in Small Animals

Depending on the type of study being designed, there are different safety issues to consider. Some optical imaging modalities involve directly exposing cerebral tissue via thinning of the skull or creation of a cerebral window, after which intravascular labels can be monitored as close to the surface as possible [61]. Because of the invasiveness required, in addition to offering comparatively small fields of view due to minimal penetration of tissue, they are commonly employed in small animal preclinical studies [63, 69]. Alternatively, other optical approaches such as fluorescence microscopy offer the ability for repeated imaging longitudinally in the same animals, which circumvents two major problems: (1) needing to sacrifice animals after critical time intervals in a study and (2) using different cohorts of animals for different time points [89]. In this way, noninvasive imaging techniques may allow for longer experiments over a span of several days, weeks, or months with minimal interruption to the animals whilst monitoring dynamic processes like neurovascular remodelling after ischaemic stroke.

Dose-equivalent radiation is another concern. The performance of *µ*CT is influenced by the X-ray source utilised. In preclinical applications, *µ*CT involves significantly lower photon output than that used in clinical CT scanners. Long (e.g., around 50 min) average scan durations may thus be required for preclinical *µ*CT to achieve comparable noise levels to those in clinical CT [90]. Maintaining recovery anaesthesia, restricting movements, and minimising ionising radiation doses over longer periods then become major procedural concerns [39]. The effects of large doses of radiation on such small animals must also be considered. However, a recent study that evaluated X-ray doses and their corresponding biological effects in experimental animals that underwent cone-beam *µ*CT scans demonstrated no significant radiation damage in the animals used [91].

Imaging system design may sometimes dictate animal handling. For example, in MRI studies, no items comprising ferromagnetic metals should be present within an MRI suite; this inflicts limitations on equipment that may be needed for small animals in a given investigation, such as monitoring probes, catheters, needles, metal-containing sutures, or implants. Although shielding from magnetic penetration may be implemented for certain tools, MRI-compatible alternatives are available for instruments that must be present within the MRI suite and within close proximity to the MRI unit [92].

Functional imaging modalities, which reveal physiological changes in response to experimental interventions, mandate stable homeostasis. The administration of anaesthesia may influence key parameters, such as blood volume, flow, and oxygenation; therefore, these changes must be acknowledged and compensated for when administering anaesthetics, analgesics, fluids, body temperature control, blood pressure regulation, or artificial ventilation [27, 93–95]. Other considerations when using small animal models arise simply from their small size, which imposes the need for specialised equipment. In experimental models, it is important for stroke to be stimulated under sterile and hygienic conditions to minimise the occurrence of wound infections. It is crucial to verify that any neuroinflammation that is identified using imaging techniques stems from ischaemia, rather than inadequate aseptic methods [96]. Administering sufficient anaesthesia for the duration of the imaging scan, sustaining metabolic homeostasis via dedicated monitoring equipment, and restricted access to the animal during the imaging scan are also prominent aspects that require consideration to ensure that the instruments utilised do not generate image distortions or hazards to the animals or researchers [92].

### 4.8. Potential Limitations in Imaging Vascular Remodelling

Improving the interpretation of imaging data is an active field of research to overcome the functional limitations present in various imaging techniques. For example, PET produces high-resolution images that are subject to background noise in the data that must be corrected for. Imaging vascular changes using contrast-enhanced methods experiences similar issues. Contrast-enhanced imaging methods rely on the distribution of molecules throughout the vasculature to be visualised. In ischaemic injury, vessel disruption directly affects the distribution of contrast. Acutely, this absence of contrast agents may contribute to the diagnosis of ischaemia; however, in long-term settings, the remodelling itself may contribute to the absence of contrast agents by means of vessel occlusion, haemostasis, or subthreshold quantities of contrast in these vessels, thus limiting detection on image acquisition. This disruption in blood flow is certainly not uncommon in ischaemic injury, and this phenomenon has been previously demonstrated with the use of ultrafast Doppler angiography to study coronary blood flow dynamics [97]. Cerebral blood volume is another concern when assessing neurovascular remodelling because an increase can represent either neoangiogenesis or vessel dilatation [14]. Lack of definition is also an issue to consider when imaging vasculature after injury. The damaged vessels may also allow the contrast agent to leak into the interstitial space around the vessels, which can inevitably distort the resulting images obtained by generating blurred images [98]. Thus, in such cases, certain types of noise in the data could potentially represent vascular leakage or, comprising a more positive outlook, could provide information on changes in postinjury blood flow dynamics.

One overarching aspect that may be worth considering when interpreting images from the translation of preclinical research to its application to humans is the age of animal models used. In general, younger animal models are used for research purposes simply because they are easier to manage and incur lower laboratory costs; older animals would need to be maintained for several months until the equivalent “old age.” However, ischaemic injury in the form of stroke is a much more common occurrence in elderly individuals amongst humans. The ischaemic penumbra also varies in measurements of size and its incorporation into the ischaemic core over time between young and old animals [96]. As a result, it may be worth imaging small animals at various time points into “older” age to inform the interpretation of imaging results in preclinical models. Whether the physiology and pathology of younger preclinical models provide a sufficient platform to develop effective treatments in older humans is a point to consider in future research.

### 4.9. Future Prospects

The current trends in preclinical imaging are progressing towards synergistic methods, allowing for the simultaneous extraction of the beneficial characteristics of each individual modality, a broader range of information that can be obtained from resulting images, and thus, optimisation of diagnosis. It is difficult to establish a single imaging technique that is optimal in all cases, as this will likely require a trade-off between different parameters; instead, multimodal techniques can be employed to take advantage of the most optimal parameters. Furthermore, with the data obtained from multiple imaging techniques, a greater number of parameters may be integrated into or used to confirm mathematical models to shed further light on physiological and pathological information [69, 73].

**Figure 8:**
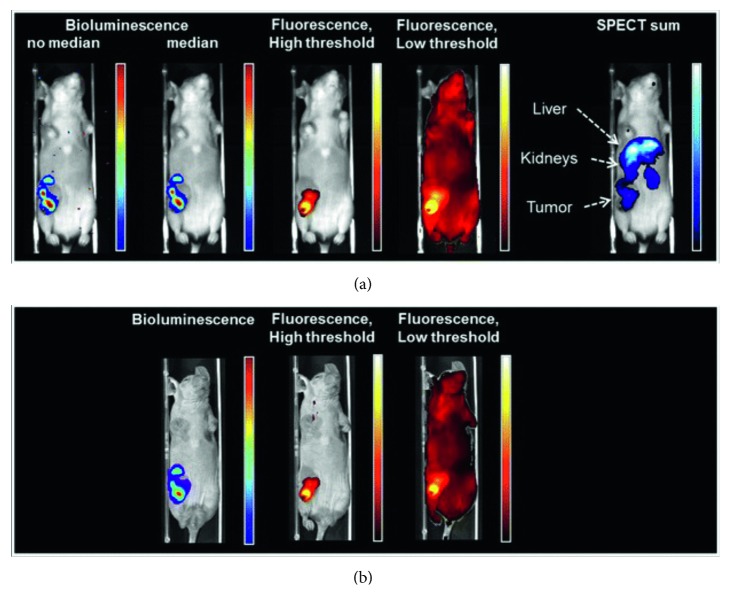
Images of a mouse with a 4T1-luc + tumour. Bioluminescence images were acquired following D-luciferin injections; fluorescence and SPECT images were acquired following multimodal tracer [^111^In]-RGD-MSAP injections. (a) U-SPECT-BioFluo bioluminescence images with and without median filter applied, fluorescence images with high and low threshold applied, and SPECT image. (b) IVIS bioluminescence and fluorescence images. SPECT, single-photon emission computed tomography; IVIS, in vivo imaging system. This figure is published in [99], which is distributed under the terms of the Creative Commons Attribution 4.0 International License (http://creativecommons.org/licenses/by/4.0).

A multimodal imaging system with the ability to perform SPECT, bioluminescence imaging, and fluorescence imaging—referred to as the U-SPECT-BioFluo system—was introduced to elucidate physiological and anatomical information within the body, specifically to target tumours. To achieve this, mouse models were administered with both a fluorescent optical dye and radioactive agent to visualise their molecular-level interactions within the body. This imaging system was originally introduced with specific relevance to study tumourigenic angiogenesis, and similarly, such a system could be employed to study the vascular changes (which also involve angiogenesis, amongst other cellular processes) following ischaemic injury (Figure 8) [99]. On a similar note, another group developed a hexamodal imaging system that exploited a unique porphyrin-phospholipid-coated type of nanoparticle. These nanoparticulate agents can be utilised to retrieve information from six different imaging modalities that comprise this system: upconversion, fluorescence, photoacoustic, PET, CT, and Cherenkov luminescence imaging [100]. Highly integrated systems such as this show great promise and feasibility for providing information that may not be achievable with single or dual imaging modalities. Multimodal neuroimaging hence represents the foundation for advancing postischaemic restorative therapies. Combinatorial techniques can ultimately be implemented to achieve a more needs-based and personalised approach in medicine and healthcare.

**Figure 9:**
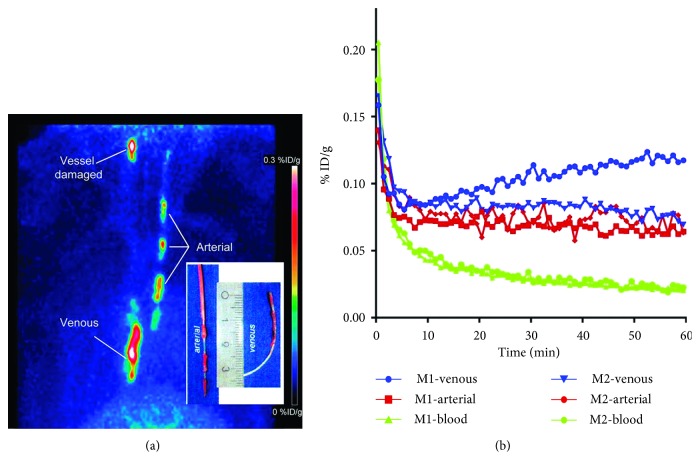
(a) [^18^F]GP1 PET image of cynomolgus monkeys with arterial and venous catheters that were inserted in the right carotid artery and vena cava, respectively (maximum-intensity projection 0–60 min). Both arterial and venous thrombi exhibited tracer uptake. (b) [^18^F]GP1 time-activity curves of thrombus uptake and blood clearance in monkeys 1 (displayed in (a)) and 2. [^18^F]GP1, [^18^F]glycoprotein-1; PET, positron emission tomography; M1, monkey 1; M2, monkey 2; %ID/g, percentage injected dose per gram. This research was originally published by Lohrke et al. [101].

Novel tracers that target different molecular pathways and can be utilised with existing imaging systems are also continuously under investigation. For example, the newly characterised PET tracer [^18^F]glycoprotein-1 ([^18^F]GP1) has been demonstrated to elucidate regions of platelet aggregation and thrombi in cynomolgus monkeys. Small venous and arterial thrombi were clearly and simply visualised in real time, and the tracer exhibited rapid blood clearance, raising the possibility of imaging ischaemia-related vascular changes in humans using [^18^F]GP1 (Figure 9) [101]. In addition, another group demonstrated the feasibility of a dual optical and PET/CT tracer for (a) noninvasive in vivo imaging of activated macrophages and vascular inflammatory activity in atherosclerotic plaques in mouse models and (b) visualisation of activated macrophages in human carotid plaque tissues, based on the activity of cysteine cathepsins [102].

Developments in MRI systems that may be useful in cases of ischaemia are also worth mentioning here. Four-dimensional (4D) flow MRI has been demonstrated to be useful for the evaluation of neurovascular haemodynamics and cerebral blood flow [103, 104]. Although long acquisition times (approx. 5–20 min) and a spatial resolution that is insufficient to capture small-vessel haemodynamics represent considerable technical limitations, 4D flow MRI comes with the benefit of evading the necessity for gadolinium contrast agents [105]. To achieve superior image contrast, MRI-based quantitative susceptibility-weighted imaging offers a way to facilitate the delineation of neural structures with the added advantage of quantification of magnetic properties in the brain [83]. Ultimately, preclinical imaging is performed with the aim of translating findings to clinical practice. Given the fact that the most common preclinical imaging techniques are MRI followed by PET, as identified in this systematic literature review, their direct combination in dedicated PET/MRI systems can prove to be advantageous for providing complementary functional and structural data. This would be advantageous for studying ongoing vascular changes; however, technical developments are warranted to facilitate accurate quantification and improved spatiotemporal resolution [106, 107].

Through its wide-ranging applications, preclinical imaging generously lends itself to translational approaches from small animals to humans. The ability to visualise tissues and organs influences the precision and accuracy of diagnosis, staging, treatment planning, and treatment response evaluation. MRI, PET, SPECT, CT, and ultrasound are commonly used in clinical practice for various imaging purposes. Although optical imaging modalities are relatively not as frequently implemented in the clinic, they are increasingly garnering attention for clinical translation [108]. The Network for Translational Research: Optical Imaging in Multimodal Platforms is a programme dedicated to developing, optimising, and validating imaging methods for rapid clinical translation. In particular, this network focusses on early-stage imaging using multiple common modalities in combination with an optical imaging modality; this combination may facilitate a quicker progression of optical imaging modalities to clinical trials [109, 110].

Furthermore, multidisciplinary collaboration can prove to enhance the value of imaging technology in translating preclinical animal models to clinical settings. For example, point-of-care technology is emerging as the future direction for clinical applications of imaging to enhance patients' experiences and decrease associated expenses. The most significant advantage of point-of-care medical imaging is the capacity to provide instant information to guide immediate clinical management decisions, as it acts as a first-line tool to screen for acute medical problems, particularly in resource-limited settings [60]. Ultimately, optimal medical and molecular imaging methods may contribute to more accurate diagnoses at earlier disease stages and prior to and during surgical operations; in due course, these methods are anticipated to enhance therapeutic outcomes in patients.

## 5. Conclusions

The field of neuroimaging has augmented our understanding of the mechanisms of ischaemic trauma, and a variety of imaging techniques are particularly helpful for observing subsequent vascular remodelling within the brain. Appropriate combinations of imaging modalities incorporating biomarkers of blood flow, energy fluctuations, and neurovascular breakdown can potentially elucidate optimal perspectives of postischaemic reorganisation.

This review is a systematic study and analysis of the literature published between 2008 and 2018 regarding preclinical in vivo imaging biomarkers for postischaemia neurovascular remodelling. The search identified 35 peer-reviewed research articles, which predominantly reported on the use of MRI or PET, although other imaging modalities were also utilised. There remain critical gaps in our knowledge within the field of restorative therapy for neurological pathologies, such as traumatic brain injury and ischaemia; amongst them, being able to detect vascular remodelling noninvasively and observe its evolution through stages of vascular recovery is an important aspect. The push towards molecular imaging has promoted the development of biological target-specific tracers to reveal the physiological and pathological mechanisms within the brain. Concurrently, the prospect of multimodality clinical imaging with its unification of anatomical and functional information holds superior potential in expanding our understanding of neurovascular organisation and disease activity.

## Figures and Tables

**Table 1:** Literature search strategy.

Initial search terminology	Synonyms and related terminology	Truncations and wildcards
ischaemic	ischaemia low blood low blood flow low oxygen hypoxia stroke	isch?emia ischaemi^*∗*^ ischemi^*∗*^ hypoxi^*∗*^

neuro	brain cerebral neurological	neuro^*∗*^ cerebro^*∗*^

vascular	vasogenic blood vessels arteries	vascular^*∗*^ arteri^*∗*^

remodelling	rearrangement reorganisation	remodel?ing remodel^*∗*^ reorgani?ation

imaging	scanning observing assessing measuring monitoring detecting	imag^*∗*^ scan^*∗*^ observ^*∗*^ assess^*∗*^ measur^*∗*^ monitor^*∗*^ detect^*∗*^

The initial search terminology stemmed from the wording of the overall aim of the review. The inclusion of alternative terminology and truncation and wildcard operators helped to uncover a wider range of papers from the databases. Some searches were also conducted with the exclusion of “remodelling” to assess whether imaging methods could potentially be implemented for this purpose.

**Table 2:** Literature search results and extracted data.

Reference	Method and application (models)		Imaging parameters	Contrast agent or molecular probe	Safety considerations for clinical translation	Applications
Bosomtwi et al. [14]	MRI to observe poststroke vascular changes (rats)		FOV: 32 mm	Feridex	Noninvasive	Tissues can be monitored long term through stages of angiogenesis enabling evaluation of vascular remodelling

Bosomtwi et al. [15]	MRI in combination with LSCM to visualise postischaemic changes in vasculature (rats)		FOV: 32 mm	MIONs	Noninvasive; high doses of intravascular agent are required	LSCM can be used to validate MRI data; poststroke vascular remodelling can be three-dimensionally quantified

Brunner et al. [16]	fUS to measure postischaemic cerebral blood volume (rats)		Resolution: 100 *μ*m, FOV: 12.8 × 9 mm^2^, duration: approx. 3 min	None	No contrast agent injections are required	Stroke longitudinally studied across all stages; can image whilst in motion, as the probe is implanted on the head

Cai et al. [17]	PET to observe VEGFR expression in poststroke angiogenesis (rats)		—	^64^Cu-DOTA-VEGF_121_	—	Some cellular VEGFRs may be visualised, resulting in the potential to observe poststroke reorganisation and plasticity

Deddens et al. [18]	MRI to detect vascular remodelling after cerebral ischaemia (mice)		FOV: 1 × 1.2 × 2 cm^3^	PECAM-1-targetted FeO_*x*_ microparticles	—	PECAM-1 can be used to assess poststroke vascular remodelling

Ding et al. [19]	MRI to visualise poststroke cerebral angiogenesis (rats)		FOV: 32 × 32 × 16 mm^3^	Gd-DTPA	Noninvasive	Detect angiogenesis and determine the temporal profile of angiogenic processes

Errico et al. [20]	Ultrafast US localisation microscopy to visualise neurovasculature and quantify haemodynamic characteristics (rats)		Resolution: 12.5 × 2.5 × 1 *μ*m^3^	Inert perfluorocarbon-filled microbubbles	Microbubbles are clinically approved contrast agents	Even slight haemodynamic changes in neurovasculature can be monitored; the resolution can be enhanced by localising microbubbles directly from radiofrequency data; motion correction algorithms needed

Figueiredo et al. [21]	CTA	To observe cerebral vascular anatomy and blood flow (mice)	Resolution: 16^3^ *μ*m^3^, duration: 20–40 s	Iomeprol	Injection of contrast agent is required	Can detect changes in the diameter of vasculature
	MRA		Resolution: 31 × 31 × 93 *μ*m^3^, FOV: 12 × 16 mm^2^, duration: 58 min	None	No ionising radiation	—
	Digital subtraction angiography		Resolution: 14 × 14 *μ*m^2^, duration: 3 s	Iomeprol	Low injection volume and dose of radiation, although much more invasive than CTA and MRA	Can detect changes in intracerebral blood flow

Gramer et al. [22]	PET, LSI, and RGB reflectometry to measure CBF, blood oxygenation, and glucose metabolism (rats)		Resolution (PET): 1.3 mm (FWHM), FOV: 12 × 7 mm^2^	[^18^F]FDG	Thin-skull preparation is required	Can be used to quantify metabolic activity of neurovasculature in real time making it suitable for studying pathological conditions. Partial volume is an issue

Horton et al. [23]	Triphoton fluorescence microscopy to visualise hippocampal vasculature (mice)		Resolution: 4.4 *μ*m (axial, FWHM)	Dextran-coupled Texas Red dye	—	Overcomes the limitations of two-photon microscopy, such as signal-to-background ratio of excitation in scattering tissues and lack of fluorescent labels that can be used

Howles et al. [24]	Contrast-enhanced MRA to visualise neurovasculature (mice)		Resolution: 52 × 52 × 100 *μ*m^3^, FOV: 20 × 20 × 8 mm^3^, duration: approx. 12 min	SC-Gd liposomal nanoparticles	—	SC-Gd allows for high contrast-to-noise ratio; useful to visualise very small vascular structures

Hu et al. [25]	Optical-resolution PAM to study micro-haemodynamic activities (rodents)		Resolution: 5 × 15 *μ*m^2^	—	Noninvasive	Can help quantify changes in metabolic parameters

Huang et al. [26]	MRI to assess vascular reactivity and functionality during postischaemic proangiogenic vascular remodelling (rats)		FOV: 2.56 × 2.56 cm^2^	—	—	Anaesthesia protocols must be optimised to minimise physiological disturbance

Jimenez-Xarrie et al. [27]	MRI to assess postischaemic cerebrovascular damage		FOV: 32 × 32 mm, duration: 9 min 17 s	None	Isoflurane anaesthesia can affect stroke outcomes and evaluation of vascular changes	Long-term vascular consequences of ischaemia with coincident hypertension can be studied

Kolodziej et al. [28]	SPECT to study CBF (mice)		Resolution: 0.7 mm (FWHM), FOV: 20.9 mm (axial), duration: approx. 2 h	^99m^Tc-HMPAO	^99m^Tc-HMPAO is lipophilic and is quickly cleared from the plasma	Uses pinhole imaging for higher resolution

Lake et al. [29]	MRI to assess poststroke brain morphology and vascular function (rats)		Resolution: 0.1 × 0.1 mm^2^, duration: <12 min	—	Propofol anaesthesia induces 20–60% regional vasoconstriction, which may influence vascular studies	Functional MRI can be used to measure resting blood flow and cerebrovascular reactivity; structural MRI may have limited sensitivity to detect subtle changes in tissue morphology

Lecoq et al. [30]	Two-photon phosphorescence lifetime microscopy to measure the partial pressure of oxygen and blood flow (mice)		Resolution: <1 *μ*m (lateral)	Phosphorescent nanoprobe PtP-C343	Minimally invasive; the probe is neither toxic nor phototoxic	Oxygen gradients in microvascular networks can be distinguished; this is particularly useful for postischaemia imaging

Letourneur et al. [31]	Two-photon laser scanning microscopy to longitudinally image vascular development (mice)		Duration: 50–150 s	Fluorescein-conjugated dextran and Texas Red-dextran	Requires thinning of the skull; head must be immobilised	Can longitudinally image the same areas over many days; can measure flow dynamics over time in relation to changes in vessel diameter

Li et al. [32]	LSI to study neurovasculature (rats)		Resolution: 6.7 × 6.7 *μ*m^2^	None	Requires thinning of the skull	Different circulatory dynamics can be observed at different spatial locations

Liao et al. [33]	Functional PAM to study functional changes in total haemoglobin concentration, cerebral blood volume, and haemoglobin O_2_ saturation in cerebral blood vessels (rats)		Resolution: 36 × 65 *μ*m^2^	None	—	Can be complemented with other imaging modalities for label-free visualisation of neurovasculature

Lin et al. [34]	3D Δ*R*_2_-based microscopy of MRA to visualise poststroke changes in neurovasculature (rats)		Resolution: 54 × 54 × 72 *μ*m^3^, FOV: 2.8 × 2.8 × 1.4 cm^3^, duration: 76 min	MIONs	Greater magnetic fields may be needed to visualise smaller vessels	Can simultaneously visualise microvascular morphology and reveal physiological properties of microvascular cerebral blood volume

Luckl et al. [35]	LSI and imaging of intrinsic signals to study CBF dynamics during ischaemia (rats)		Resolution: 140 *μ*m every 2 s, FOV: 5 × 5 mm^2^	Erythrosin B dye	Requires thinning of the skull for better observation	Vascular changes in metabolism can be quantified

Miao et al. [36]	LSI to study angiogenesis (rats)		FOV: 4.7 × 4.7 mm^2^	None	Requires thinning of the skull	CBF under various pathological states can be analysed, and smaller vessels can be enhanced; results can be affected by motion artefacts

Nagaraja et al. [37]	MRI to visualise poststroke changes in the BBB (rats)		FOV: 32 mm	Gd-DTPA and Gd-DTPA linked to bovine serum albumin and Evans blue dye	Noninvasive	Different measurements are obtained with different contrast agents; quantifying BBB permeability can help in understanding the progression of ischaemic injury

Sakadžić et al. [38]	Two-photon phosphorescence lifetime microscopy to measure partial pressure of oxygen in cortical microvasculature under hypoxic conditions (rats, mice)		—	Phosphorescent nanoprobe PtP-C343	Minimally invasive with low doses of the probe required; no detected leakage of the probe into interstitial spaces	The partial pressure of oxygen can be simultaneously assessed at various positions and depths, making it more feasible to functionally study transient changes in oxygen levels

Schambach et al. [39]	Volume-CTA to visualise cerebral vessels (mice)		Duration: 40 s	Iodinated contrast agent	Large dose of contrast agent is required	Changes in vessel diameter can be monitored

Schroeter et al. [40]	PET	To observe postischaemic vascular changes (rats)	Duration: up to 60 min	[^18^F]FDG and [^11^C]PK11195	Noninvasive	Characterise neuroinflammation and metabolic disruptions repeatedly over time.
	MRI		FOV: 3.0 cm	—	Noninvasive	Can help localise areas of infarction

Seo et al. [41]	Contrast-enhanced *μ*CT to visualise poststroke changes in cerebral vasculature (rats)		Duration: approx. 2 min	Iopromide	High doses of iodinated contrast agent are needed	Images are subject to blurring due to physiological motion

Stein et al. [42]	PAM to study blood oxygenation dynamics of hypoxic cerebral vasculature (mice)		Resolution: 70 × 54 *μ*m^2^	None; monitors “endogenous” haemodynamics	Noninvasive	Single blood vessels can be noninvasively assessed in real time

Struys et al. [43]	PET	To characterise acute and long-term vascular and metabolic effects of unilateral common carotid artery occlusion (mice)	Resolution: 1.35 mm (transaxial, FWHM), duration: 10 min	[^15^O]H_2_O and [^18^F]FDG	—	Can be used to monitor short-term/long-term perfusion and vascular remodelling in ischaemic stroke models
	MRI		Resolution: 98 × 98 *μ*m^2^, FOV: 2.5 × 2.5 cm^2^	—		

Tsukada et al. [44]	PET to study postischaemic changes (monkeys)		Duration: 91 min	[^18^F]flurpiridaz and [^18^F]BCPP-EF	Surgical procedures are invasive and require anaesthesia	Study metabolic properties and distinguish inflammatory processes

Yanev et al. [45]	Steady-state contrast-enhanced MRI to assess the changes in cerebral blood volume and microvascular density after transient stroke (rats)		FOV: 30 × 30 mm^2^, duration: approx. 135 min	Ultrasmall iron oxide particles	—	Changes in cerebral blood volume and microvascular density can be observed at least 3 months after stroke; only perfused (and therefore functional) vessels can be detected

Yoon et al. [46]	Multiphoton luminescence to visualise morphological changes in cortical vasculature over time (mice)		—	PEG-GNPs	PEG-GNPs are highly biocompatible	Long circulation time of PEG-GNPs enables vascular imaging for several hours, making them suitable to observe remodelling

Yu et al. [47]	Spectral Doppler OCT to quantitatively assess dynamic blood flow before and after stroke (mice)		Duration: approx. 20 min	Rose bengal	—	Mimics ischaemic conditions by reducing CBF in microvasculature; the pulsatility of CBF is quantified; changes in heart rate due to anaesthesia wearing off and being readministered must be considered

Zhang et al. [48]	SR-PCI to visualise neural microvasculature (rats)		Resolution: <10 *μ*m, FOV: approx. 3 mm	None	High doses of ionising radiation	Vascular architecture and volume can be visualised and quantified; FOV is limited

External sources were not consulted to fill in missing details at this stage in the research. Abbreviations: FOV, field of view; MRI, magnetic resonance imaging; LSCM, laser scanning confocal microscopy; MION, monocrystalline iron oxide nanoparticle; fUS, functional ultrasound; PET, positron emission tomography; VEGFR, vascular endothelial growth factor receptor; PECAM, platelet endothelial cell adhesion molecule; Gd-DTPA, gadolinium-diethylenetriaminepentaacetate; US, ultrasound; CTA, computed tomography-angiography; MRA, magnetic resonance angiography; LSI, laser speckle imaging; RGB, red-green-blue; CBF, cerebral blood flow; FWHM, full width at half maximum; [^18^F]FDG, [^18^F]fluorodeoxyglucose; SC-Gd, surface-conjugated gadolinium; PAM, photoacoustic microscopy; SPECT, single-photon emission computed tomography; ^99m^Tc-HMPAO, ^99m^Tc-hexamethylpropyleneamineoxime; BBB, blood-brain barrier; *μ*CT, microcomputed tomography; PEG-GNP, polyethylene glycosylated gold nanoparticle; OCT, optical coherence tomography; SR-PCI, synchrotron radiation phase contrast imaging.
